# Mind-Body Intervention for Stress and Hypertension in Black Female Dementia Family Caregivers: A Feasibility Study

**DOI:** 10.1093/geroni/igaf122.677

**Published:** 2025-12-31

**Authors:** Kathy Wright, Ingrid K Richards Adams, Nathan Helsabeck, Karen Moss, Donya Nemati, Christopher Nguyen, Maryanna Klatt

**Affiliations:** The Ohio State University, Powell, Ohio, United States; The Ohio State University, Columbus, Ohio, United States; The Ohio State University, Columbus, Ohio, United States; The Ohio State University, Blacklick, Ohio, United States; Ohio State University, Columbus, Ohio, United States; The Ohio State University, Columbus, Ohio, United States; The Ohio State University, Columbus, Ohio, United States

## Abstract

Black females caring for family members living with Alzheimer’s disease and related dementias (ADRD) face high risks of caregiving stress and hypertension, leading to reduced quality of life and cardiovascular disease. While some interventions address ADRD caregivers’ stress and quality of life, gaps remain in targeting chronic caregiving stress and hypertension self-care. This Stage I (1R21AG077069) pilot study was conducted to determine the feasibility and acceptability of Mindfulness in Motion plus the Dietary Approaches to Stop Hypertension (MIM DASH) for Black female ADRD family caregivers. Participants (N = 28) were randomized to either MIM DASH or Alzheimer’s Association Caregiver Training (attention control) for eight weekly, 1.5-hour group sessions via Zoom Communications, Inc (video and telephone access). After completion of the intervention, both groups received eight weekly calls for two months, followed by one monthly call for four months. The intervention demonstrated high feasibility, with a 93% enrollment rate and 86% and 93% retention rates at three- and nine-month follow-ups, respectively. Participants in the MIM-DASH group attended an average of 6.29 sessions compared to 4.86 for the attention control group. Usability scores for MIM-DASH participants were high (mean 50.31), and satisfaction ratings averaged 8.77 out of 10. Credibility ratings were similarly robust, with a mean score of 41.23 out of 45 for the intervention group. Fidelity was maintained with minor deviations observed. MIM DASH was feasible and acceptable for Black female caregivers. Findings support the delivery of a Stage II efficacy trial.

